# Curcumin: a therapeutic strategy in cancers by inhibiting the canonical WNT/β-catenin pathway

**DOI:** 10.1186/s13046-019-1320-y

**Published:** 2019-07-22

**Authors:** Alexandre Vallée, Yves Lecarpentier, Jean-Noël Vallée

**Affiliations:** 1 Diagnosis and Therapeutic Center, Hypertension and Cardiovascular Prevention Unit, Hotel-Dieu Hospital, AP-HP, Université Paris Descartes, 1 place du Parvis de Notre-Dame, Paris, France; 2 Centre de Recherche Clinique, Grand Hôpital de l’Est Francilien (GHEF), 6-8 rue Saint-fiacre, 77100 Meaux, France; 3 Centre Hospitalier Universitaire (CHU) Amiens Picardie, Université Picardie Jules Verne (UPJV), 80054 Amiens, France; 4 Laboratoire de Mathématiques et Applications (LMA), UMR CNRS 7348, Université de Poitiers, Poitiers, France

**Keywords:** Curcumin, Cancer, WNT pathway, Inflammation, Oxidative stress, PPARγ

## Abstract

Numerous studies have presented that curcumin could have a positive effect in the prevention of cancer and then in tumor therapy. Several hypotheses have highlighted that curcumin could decreases tumor growth and invasion by acting on both chronic inflammation and oxidative stress. This review focuses on the interest of use curcumin in cancer therapy by acting on the WNT/β-catenin pathway to repress chronic inflammation and oxidative stress. In the cancer process, one of the major signaling pathways involved is the WNT/β-catenin pathway, which appears to be upregulated. Curcumin administration participates to the downregulation of the WNT/β-catenin pathway and thus, through this action, in tumor growth control. Curcumin act as PPARγ agonists. The WNT/β-catenin pathway and PPARγ act in an opposed manner. Chronic inflammation, oxidative stress and circadian clock disruption are common and co-substantial pathological processes accompanying and promoting cancers. Circadian clock disruption related to the upregulation of the WNT/β-catenin pathway is involved in cancers. By stimulating PPARγ expression, curcumin can control circadian clocks through the regulation of many key circadian genes. The administration of curcumin in cancer treatment would thus appear to be an interesting therapeutic strategy, which acts through their role in regulating WNT/β-catenin pathway and PPARγ activity levels.

## Background

The complex process of cancer can be defined in terms of three stages: initiation, promotion and progression [1–3]. Many cancers are initiated by chronic inflammation, involving numerous physical, chemical and biological determinants [4]. Several studies have examined the relationship between chronic inflammation and cancer [5, 6] and indeed have highlighted the promising role of anti-inflammatory treatments for cancer [7]. Chronic inflammation is responsible for the different stages observed in cancers, such as invasion, angiogenesis, proliferation and metastasis [8–10].

In parallel, oxidative stress promotes DNA damages in cancers [11]. Since few years, the combination formed by oxidative stress and chronic inflammation is involved in the initiation of cancer [12]. Reactive oxygen species production (ROS) is increased by the activation of inflammatory factors [13] and thus also participates in the process of invasion, proliferation, angiogenesis and then metastasis [14]. The canonical WNT/β-catenin pathway controls several other pathways involved in development and tissue homeostasis. This pathway is regulated from transcription-level regulations to post-transcriptional modifications. An aberrant WNT/β-catenin pathway is generally observed in cancers and leads to inflammation and oxidative stress [12, 15].

The recent therapeutic strategies in cancer are associated with several limitations, such as high risk of relapse, drug resistance, poor outcomes and unavailability of therapy. However, plants are the site of promise sources of bioactive natural components [16]. These natural compounds could be interesting and novels strategies in therapy. Curcumin (1,7-bis (4-hydroxy-3-methoxyphenyl)-1,6-heptadiene-3,5-dione) is a natural product which occurs polyphenolic phytochemical properties from the rhizome of the *Curcuma longa* L. [17]. Curcumin has been discovered in 1815 by Vogel and Pelletier [18]. Its yellow-colored hydrophobic component is traditionally used in Asian countries for its several properties against pathophysiological states including anticancer [19]. Several targets of curcumin have been shown to have chemotherapeutical properties. Curcumin use may have a major role in the control of inflammation, angiogenesis, metastasis and proliferation [20]. Curcumin can downregulate numerous pathways, such as nuclear factor-ϰ B (NF-ϰB), cyclooxygenase-2 (COX-2), and the canonical WNT/β-catenin pathway [20].

The chronic inflammatory microenvironment of tumors could be targeted by curcumin. It is well known that the human body is capable of self-healing after a short-term inflammatory response, but a long-term chronic inflammation could lead to initiation of the cancer process. Many studies have shown that inflammatory factors (including interleukins, TNF-α, NF-ϰB) and the ROS production-induced inflammation infiltrate the inflammatory microenvironment leading to DNA damages and ultimately initiation of cancer [21]. By acting on several signaling pathways, especially the WNT/β-catenin pathway, curcumin can have anticancer effect by inhibiting chronic inflammation and oxidative stress [22].

Curcumin acts as peroxisome proliferator-activated receptor gamma (PPARγ) agonists and thus downregulate the aberrant WNT/β-catenin pathway observed in cancers [23]. PPARγ agonists offer an interesting therapeutic solution in cancers by acting on both oxidative stress and inflammation [24, 25]. Indeed, in several tissues, overactivation of the canonical WNT/β-catenin pathway induces the downregulation of PPARγ, while PPARγ activation induces inhibition of canonical WNT/β-catenin pathway. In mainly cancers, the canonical WNT/β-catenin pathway is increased while PPARγ is downregulated [12].

In parallel, dysregulation of circadian rhythms (CRs) has been observed in cancers [26]. This dysfunction leads to the upregulation of the canonical WNT/β-catenin pathway contributing to cancer initiation. PPARγ can control CRs by regulating many key circadian genes, like Bmal1 (brain and muscle aryl-hydrocarbon receptor nuclear translocator-like 1) [27] and then can target WNT pathway [28].

This review focuses on the interest of use curcumin in cancer therapy by acting through the opposed interaction between the canonical WNT/β-catenin pathway and PPARγ to repress chronic inflammation and oxidative stress, and to control circadian rhythms.

### Curcumin: a new agent for therapeutic strategy in cancers

Phytotherapy has claimed importance globally in cancer therapies (Table 1). Curcumin, defined as bis-α, β-unsaturated β-diketone, is a natural component well documented since 1815. Curcumin is the active compound of turmeric or *Curcuma longa* L. and presents surprising wide range of beneficial properties, such as anticancer, chemopreventive and chemotherapeutic activities [43]. The health benefits of curcumin are limited by its poor oral bioavailability which can be attributed to the poor absorption, high rate of metabolism and rapid systemic elimination from body. Indeed, curcumin is converted to its water-soluble metabolites and then excreted through urine. This metabolism is composed by two steps. First, a NADPH-dependent metabolism of reduction which comprises the reduction of the double bonds of the heptadiene-3, 5-dione structure catalyzed by NADPH-dependent curcumin reductase. Secondly, a process of conjugation has been observed with monoglucuronide via a β-glucuronidase. These two mechanisms are responsible for the low solubility and rapid metabolism of curcumin.

**Table 1:** Curcumin an anticancer agent in several tumors

Type of cancer	Actions	Type of study	References
Benign prostatic hypertrophy	Improved quality of life, reduced symptoms	Pilot product evaluation study	[29]
Breast	Inhibition cancer progression, decreased levels of VEGF	Phase I clinical trial	[30]
Chronic myeloid leukemia	Reduction of nitric oxide levels	Randomized controlled trial	[31]
Colorectal	Decrease inflammation (TNF-α), increase p53	Phase I clinical trial	[32]
Colorectal	Reduction in tumor growth	Phase I clinical trial	[33]
Colorectal	Decrease PGE2 levels	Phase I clinical trial	[34]
Colon carcinoma	Growth inhibition	Randomized controlled trial	[35]
Intestinal adenoma	Diminution of adverse effects	Randomized controlled trial	[36]
Pancreatic	Inhibition of toxicity profile of tumors	Phase II clinical trial	[37]
Pancreatic	Diminution of NF-ϰB pathway	Phase I clinical trial	[38]
Prostate	Increase survival	Randomized controlled trial	[39]
Prostate	Enhanced antiproliferative efficacy and targeting	Randomized controlled trial	[40]
Ovarian carcinoma	Increased cytotoxicity	Randomized controlled trial	[41]
Head and neck squamous cell carcinoma	Decrease inflammatory mediators	Randomized controlled trial	[42]

Even if some studies have related that pharmacokinetics of curcumin have revealed poor bioavailability [44], strong pharmacological and clinical applications have been reported for curcumin [45]. Nevertheless, some of possible ways to overcome this poor bioavailability can be counteract by centering on these aspects. Strategies can improve this bioavailability, such as phospholipid complexes, liposomes and nanoparticles. Some polymers have been used to prepare nanoformulations for curcumin drug delivery to improve its biological activity [46]. Biocompatible and biodegradable polymers are utilized in drug delivery systems due to their lower risks of toxicity [47]. Advances in liposomes formulations results in the improvement of therapy for drug-resistant tumors and in the reduction of toxicity [48]. Liposomes consist of phospholipid bilayer shells and aqueous cores resulting in a curcumin encapsulation by both hydrophobic and hydrophilic components. Other curcumin delivery systems are used, as nanogels [49], peptide and protein formulations [50] and cyclodextrin complexes [51].

### Chronic inflammation and oxidative stress in cancer process

#### Chronic inflammation

Numerous studies have presented that chronic inflammation leads to DNA damages and tissue injury [52]. Chronic inflammation impairs cell homeostasis, metabolism to initiate cancer [53]. Moreover, DNA damages involved by the chronic inflammation provides a point of origin for the initiation of malignancy sites. Several studies have well described the link between cancer and chronic inflammation [12]. Chronic inflammation activates ROS and reactive nitrogen species (RNS) production leading to DNA damages [54]. Thus, genomic instabilities are initiated by DNA damages and then cause cancer initiation. Numerous sites of common pathogenic infections are related to cancer initiation [55].

The immune system is also regulated by several inflammatory factors, such as tumor necrosis factor α (TNF-α), interleukin-6 (IL-6), vascular endothelial growth factor (VEGF) and tumor growth factor-β (TGF-β) [56]. TNF-α expression leads to DNA damages and cytokines stimulation (such as IL-17 [57]), which are responsible for tumor growth, invasion and angiogenesis [58]. Interleukins, IL6 and IL-17, activate the signal transducer and activator transcription (STAT) signaling involved in the cancer process [59].

Chronic inflammation is also responsible for an increase in cyclooxygenase 2 (COX-2, a prostaglandin-endoperoxidase synthase). Numerous cytokines (TNF-α, IL-1) activate COX-2 [60]. COX-2 stimulates ROS and RNS production [61, 62]. Nuclear factor-ϰB (NF-ϰB) stimulates several pro-inflammatory factors that activate COX-2 and inducible nitric oxide synthase (iNOS) [53]. NF-ϰB is one of the major factors involved in chronic inflammation in the cancer process [53]. Several studies have shown that NF-ϰB stimulates the expression of TNF-α, IL-6, IL-8, STAT3, COX-2, BCL-2 (B-cell lymphoma 2), metalloproteinases (MMPs), VEGF [53], and thus the ROS production [63]. Il-6 and VEGF activates STAT-3 pathway involved in proliferation, angiogenesis and metastasis [64]. Several cancers presents an over-activation of the STAT-3 pathway [65]. Furthermore, iNOS, an enzyme catalyzing nitric oxide (NO), is activated during chronic inflammation and increases p53 gene mutations [60].

#### Oxidative stress

Oxidative stress is considered as an imbalance between the production and elimination of ROS and RNS [11, 66]. ROS production is enhanced by cell damages from oxidation and nitration of macromolecules, such as RNA, DNA, proteins and lipids.

The NADPH oxidase (NOX) enzyme increases ROS production through the oxidation of intracellular NADPH to NADP^+^. Superoxide anion is then produced, and molecular oxygen phenomenon is reduced due to the transfer of electron through the mitochondrial membrane.

ROS production has a key role in numerous signaling involved in changes of microenvironment [67]. Thus, dysfunction in the respiratory chain of mitochondria is responsible for ROS production [68]. The inflammation observed, where there are damages, involves the uptake of oxygen leading in the release of ROS and its accumulation. NF-ϰB, STAT, hypoxia-inducible factors (HIF) and both activator protein-1 (AP-1) play a major role in the stimulation of this process [53]. Moreover, in a vicious circle COX-2, TNF-α, IL-6, iNOS are induced by oxidative stress [62]. NADPH-oxidase (NOX) is activated by chronic inflammation resulting in oxidative stress and alteration of the nuclear signaling [69].

### Interactions between oxidative stress and inflammation (Fig. 1)

Several researches have demonstrated the mechanism by which oxidative stress can lead to chronic inflammation, which in turn could cause cancers [11]. The imbalance caused by oxidative stress leads to damages in the signaling in cells [66]. ROS play a central role both upstream and downstream of the NF-κB and TNF-α pathways, which are the main mediators of the inflammatory response. The hydroxyl radical is the most harmful of all the ROS. A vicious circle is observed between ROS and these pathways. ROS are generated by NOX system. Moreover, the proteins modified by ROS could result in initiation of the auto-immune response to stimulate TNF-α and thus NOX [70]. Nuclear factor erythroid-2 related factor 2 (Nrf2) is mainly associated with oxidative stress in inflammation [11]. Nrf2 is a transcription factor which binds with the antioxidant response element (ARE) [71]. The protective role of Nrf2 in cancer relates to its capability to reduce inflammation and oxidative stress [72]. Several studies have shown that Nrf2 can play an anti-inflammatory role by regulating MAPK (Mitogen-activated protein kinases), NF-ϰB, and PI3K pathways [73]. Thus, Nrf2 may play a major role in reducing oxidative damages [74]. Evidence also suggested that mitochondrial dysregulation has a significant role in the cancer mechanism [11].

**Fig. 1:**
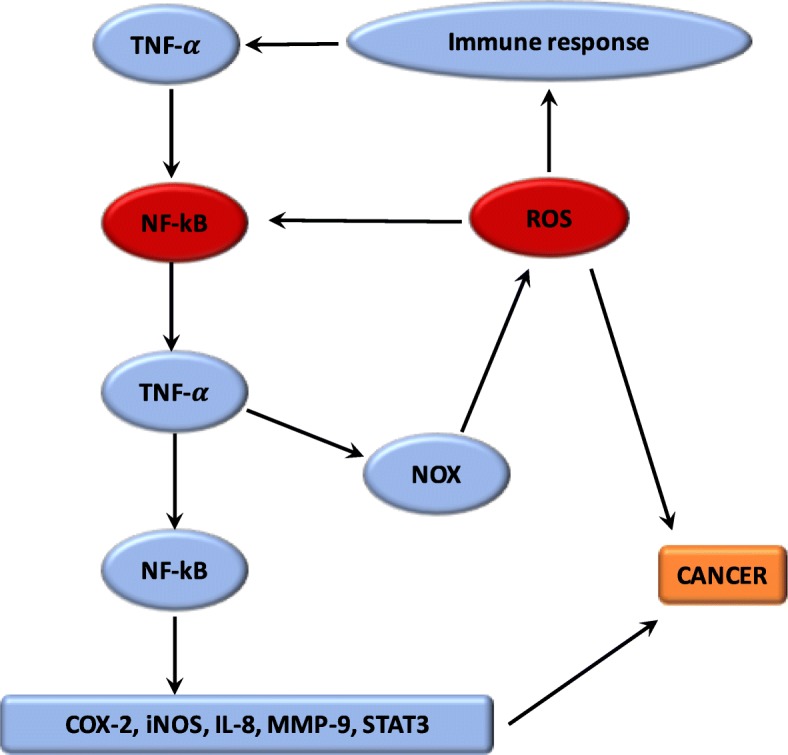
Relationship between ROS and chronic inflammation

### The WNT pathway, chronic inflammation and oxidative stress

Many studies have shown that canonical the WNT/β-catenin pathway stimulates inflammation [52]. Moreover, infection pathogens activate the WNT/β-catenin pathway enhancing thereby inflammation. ROS, stimulated by NOX, activates the canonical WNT/β-catenin pathway through the oxidization and inactivation of the nucleoredoxin (a redox-sensitive regulator), thus stimulating the cancer process [53]. ROS production leads to the activation of c-Myc, STAT, phosphatidylinositol-3-kinase (PI3K/Akt) and the inhibition of PPARγ [75]. ROS production stimulates the Akt signaling by inhibiting the phosphatase and tensin homolog deleted from chromosome (PTEN) [76]. Moreover, the canonical WNT/β-catenin pathway may thus play a major role in cancer by modulating both oxidative stress and inflammation [12].

### The canonical WNT/β-catenin pathway: a major factor in cancer process (Fig. 2)

WNT name is derived from Wingless *Drosophila melanogaster* and its mouse homolog Int. The WNT pathway is involved in several signaling and regulating pathways, such as embryogenesis, cell proliferation, migration and polarity, apoptosis, and organogenesis [77]. During the adult stage, the WNT pathway is non-activated or silent. However, during numerous mechanisms and pathologies, such as inflammatory, metabolic and neurological disorders, and cancers, the WNT pathway may become dysregulated [78]. Recent studies have used the WNT pathway for cell therapy-bioengineering processes [79].

**Fig. 2:**
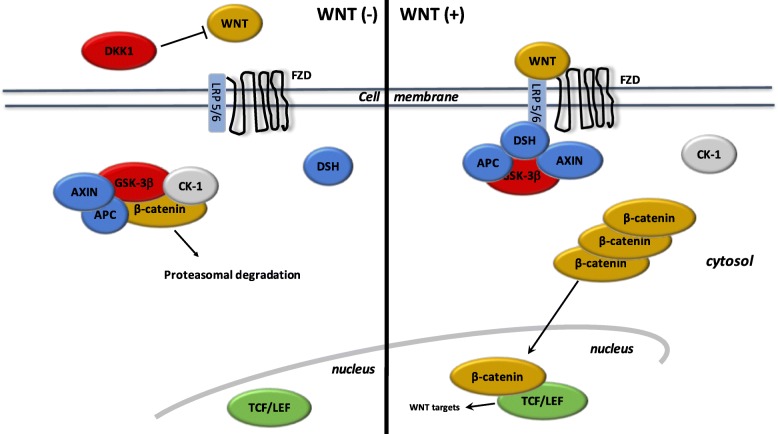
The canonical WNT/β-catenin pathway. WNT (−). Under resting condition, the cytoplasmic β-catenin is bound to its destruction complex, consisting of APC, AXIN and GSK-3β. After CK-1 phosphorylates on Ser45 residue, β-catenin is further phosphorylated on Thr41, Ser37, and Ser33 residues by GSK-3β. Then, phosphorylated β-catenin is degraded into the proteasome. Therefore, the cytosolic level of β-catenin is kept low in the absence of WNT ligands. If β-catenin is not present in the nucleus, the TCF/LEF complex cannot activate the target genes. DKK1 inhibits the WNT/β-catenin pathway by binding to WNT ligands or LRP5/6. WNT (+). When WNT ligands bind to both FZD and LRP5/6, DSH is recruited and phosphorylated by FZD. Phosphorylated DSH in turn recruits AXIN, which dissociates the β-catenin destruction complex. Therefore, β-catenin escapes from phosphorylation and subsequently accumulates in the cytosol. The accumulated cytosolic β-catenin goes into the nucleus, where it binds to TCF/LEF and activates the transcription of target genes

WNT ligands are lipoproteins that activate specific co-receptors. These WNT ligands activate the canonical WNT pathway through the action of β-catenin. WNT ligands activate Frizzled (FZD) receptors and low-density lipoprotein receptor-related protein 5 and 6 (LRP 5/6) [80]. The complex formed by extracellular WNT ligands and FZD/LRP5/6 stimulates intracellular Disheveled (DSH). This activation inactivates the destruction complex of β-catenin in the cytosol. Β-catenin accumulates in the cytosol and then translocates into the nucleus. Nuclear β-catenin interact with T-Cell factor/lymphoid enhancer factor (TCF/LEF) to stimulate gene transcription, such as c-Myc STAT, PI3K/Akt, and cyclin D1 [81].

During the “off-state” of the WNT/β-catenin pathway, WNT ligands do not bind FZD and LRP 5/6. The β-catenin destruction complex, formed by AXIN, APC (adenomatous polyposis coli) and GSK-3β (glycogen synthase kinase 3β), phosphorylates β-catenin. Thus, phosphorylated β-catenin is degraded into the proteasome.

Several WNT inhibitors inactivates the canonical WNT/β-catenin pathway. GSK-3β is the major inhibitor of the WNT pathway. GSK-3β is a neuron-specific intracellular serine-threonine kinase that regulates several signaling pathways such as inflammation, neuronal polarity and cell membrane signaling [82]. GSK-3β inhibits β-catenin cytosolic stabilization and nuclear migration. Dickkopf (DKK) and soluble Frizzled-related proteins (SFRP) are also WNT inhibitors and binds FZD, LRP5 and LRP6 [83].

#### WNT and inflammation in cancers

Positive interplay between WNT/β-catenin and NF-ϰB has been highlighted [84]. The activation of the WNT/β-catenin leads to the enhancement of IϰB-α (nuclear factor of kappa light polypeptide gene enhancer in B-cells inhibitor, α) degradation and then NF-ϰB stimulation [85]. Stimulation of the target gene, CRD-BP (Coding Region Determinant-Binding Protein, an RNA-binding protein), by activated β-catenin stabilizes mRNA of βTrCP (Beta-transducin repeat-containing protein) [86]. In colon cancer, activation of both βTrCP and CRD-BP is correlated with the stimulation of the β-catenin and NF-ϰB, leading proliferation and metastasis. In breast cancer, TLR3 activation stimulates β-catenin leading to over-activation of the NF-ϰB pathway [87]. Moreover, the β-catenin and NF-ϰB pathways stimulates each other in diffuse large B-cell lymphomas [88]. The WNT/β-catenin pathway activates COX-2, which then enhances the inflammatory response [89]. E-cadherin and GSK-3β are downregulated in melanoma cells by β-catenin signaling [90]. Concomitant GSK-3β and E-cadherin inhibition with cytosolic β-catenin accumulation leads to NF-ϰB-dependent iNOS expression in hepatic cells [91]. The WNT/β-catenin pathway stimulates its target TNFRSF19 in colon cancer, which leads to the activation of the NF-ϰB signaling [92]. Nevertheless, the observed synergistic interaction between β-catenin and NF-ϰB depends on the β-catenin-TCF/LEF link [93].

NF-ϰB over-expression inactivates GSK-3β whereas it stimulates β-catenin signaling [94, 95]. GSK-3β activation results in the downregulation of TNF-α-induced NF-ϰB stimulation in carcinoma cells [94]. IϰB is stabilized by GSK-3β activation resulting in the downregulation of the NF-ϰB pathway [95]. NF-ϰB signaling can modulate the WNT/β-catenin pathway through the use of IKKα (IϰB Kinase-α) and RelA [96]. IKKα stimulates β-catenin signaling while IKKβ inhibits β-catenin signaling [97]. IKKα activates the β-catenin/TCF/LEF link [98]. Activation of IKKα leads to the cytosolic β-catenin accumulation resulting in GSK3-β and APC inactivation [99].

#### WNT and oxidative stress in cancers

The over-activated PI3K/Akt pathway observed in the cancer process is stimulated by ROS production [100]. PTEN is the main inhibitor of the PI3K/Akt pathway [101]. NADPH oxidase and superoxide dismutase oxidize PTEN to inhibit it. Inhibition of PTEN leads to an increase in Akt activity, which enhances the phosphorylation of GSK-3β. Thus, GSK-3β inactivated by Akt does not bind β-catenin. Inactivation of PTEN activates Akt and β-catenin [102]. Moreover, ROS production participates in the stabilization of HIF-1α thereby activating glycolytic enzymes [103]. The WNT/β-catenin pathway stimulates HIF-1α by activating the PI3K/Akt pathway [104]. Although this mechanism remains unclear, recent studies have shown that ROS production stimulates the WNT/β-catenin pathway [105]. In parallel, Akt [106] and c-Myc [107] enhance ROS production.

### PPARγ in cancers

The ligand-activated transcriptional factor peroxisome proliferator receptor γ (PPARγ) is a member of the nuclear hormone receptor super family. It forms a heterodimer with retinoid X receptor (RXR), leading to a PPARγ-RXR complex which binds to specific peroxisome proliferator response element (PPRE) regions in the DNA and activating several target genes involved in fatty acid transport (FABP3), cholesterol metabolism (CYP7A1, LXRα, CYP27), glucose homeostasis (PEPCK, GyK) and lipid catabolism (SCD-1). This dimer interacts with other coactivators proteins such as PGC-1α, and induces specific genes expression [108]. Glucose homeostasis, insulin sensitivity, lipid metabolism, immune responses, cell fate and inflammation are regulated by PPARγ activation [109]. Circadian variations of blood pressure and heart rate are regulated by PPARγ through its action on Bmal1 [27]. PPARγ modulates the expression of several genes involved in inflammation, and it decreases the activity of inflammation-related transcription factors such as NF-ϰB [110]. Several studies have shown decreased PPARγ expression in association with chronic inflammation in cancers [12].

### Interplay between PPARγ and the WNT/β-catenin pathway in cancers

The action of PPARγ agonists remains unclear in cancer cells even if their role is well understood in the regulation of differentiation and stemness programs [111]. In physiological cells, PPARγ inhibits tumorigenesis and WNT signaling by targeting phosphorylated β-catenin at the proteasome through a process that involves its catenin binding domain within PPARγ. In contrast, oncogenic β-catenin counteracts proteasomal degradation by downregulating PPARγ activity, which requires its TCF/LEF binding domain [112]. In adipocyte cells, PPARγ leads to increased differentiation and a reduction in proliferation by targeting the WNT/β-catenin pathway. PPARγ binds with GSK3-β to activate the differentiation factor C/EBPα leading to the production of adiponectin [113]. PPARγ activation downregulates β-catenin at both the mRNA and protein levels to induce differentiation [114]. In metastatic prostate cancer LnCaP cells, PPARγ decreases the WNT pathway by affecting phosphorylated β-catenin in the proteasome [112, 115]. In colorectal and gastric cancer cells, PPARγ inhibits β-catenin expression, subcellular localization and downstream effectors, leading to the modulation of numerous genes, such as telomerase reverse transcriptase and Sox9, both of which are involved in cell differentiation and the survival phenomenon [116]. PPARγ agonists, by decreasing the WNT/β-catenin pathway, could be used in combination with other drugs such as inhibitors of tyrosine kinases [117], Akt [118], and MAPK cascades to maximize the antitumor and pro-differentiating effect.

### Circadian rhythms in cancers

#### Circadian rhythms: definition (Fig. 3)

Numerous biological processes in the body are regulated by the circadian “clock” (circadian locomotors output cycles kaput). The circadian clock is in the hypothalamic suprachiasmatic nucleus (SCN). CRs are endogenous and entrainable free-running periods that last approximately 24 h. Numerous transcription factors are responsible for the control of CRs. These are called circadian locomotors output cycles kaput (Clock), brain and muscle aryl-hydrocarbon receptor nuclear translocator-like 1 (Bmal1), Period 1 (Per1), Period 2 (Per2), Period 3 (Per3), and Cryptochrome (Cry 1 and Cry 2) [119, 120]. These transcription factors are subject to positive and negative self-regulation mediated by CRs [121, 122]. Clock and Bmal1 heterodimerize and thus initiate the transcription of Per1, Per2, Cry1 and Cry2 [123]. The Per/Cry heterodimer can downregulate its stimulation through negative feedback. It translocates back to the nucleus to directly inhibit the Clock/Bmal1 complex and then repress its own transcription [123]. The Clock/Bmal1 heterodimer also stimulates the transcription of retinoic acid-related orphan nuclear receptors, Rev-Erbs and retinoid-related orphan receptors (RORs). Through positive feedback RORs can stimulate the transcription of Bmal1, whereas Rev-Erbs can inhibit their transcription through negative feedback [123].

**Fig. 3:**
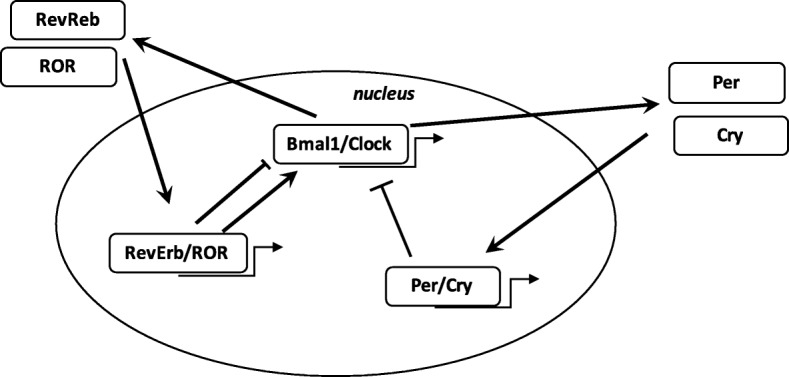
Circadian clock genes. The clock consists of a stimulatory loop, with the Bmal1/Clock heterodimer stimulating the transcription of Per and Cry genes, and an inhibitory feedback loop with the Per/Cry heterodimer translocating to the nucleus and repressing the transcription of the Clock and Bmal1 genes. An additional loop involves the RORs and RevErbs factors with a positive feedback by ROR and a negative feedback by RevErbs

#### Circadian rhythms disruption in cancers

Epidemiological and fundamental evidence supports the idea of linking circadian disruption with cancer [26]. DNA repair, apoptosis and cell cycle regulation follow circadian rhythms in humans [124]. Disruption of the CRs is associated with dysregulation in cell proliferation and thus the initiation of cancer [125]. Clock/Bmal1, Per1 and Per2 maintain the rhythmic pattern of cell proliferation and repair of DNA damage [126]. Bmal1 over-expression has been observed in cell growth of NIH 3 T3 cells [127]. Metastatic cancers present high levels of Clock or Bmal1 genes [128]. Clock over-expression is often associated with cell proliferation in colorectal carcinoma cells [129]. Bmal1 upregulation is found in certain types of pleural mesothelioma while Bmal1 knockdown is associated with reduced cell growth and induced apoptosis [130]. Bmal1 is considered as an attractive target in leukemia cells [131].

#### Circadian rhythms and inflammation

Melatonin has been used in the treatment of chronic bowel inflammation resulting in decreasing inflammation through inhibition of COX-2 and iNOS [132]. Moreover, melatonin can act on iNOS and COX-2 by suppressing p52 acetylation and transactivation [133]. Melatonin inhibits NF-ϰB and COX-2 in murine macrophage-like cells [134]. An anti-inflammatory response of melatonin has been observed through a decrease in NF-κB activity [135]. Melatonin downregulates the nuclear translocation of NF-κB, leading to an enhancement of anticancer effects in lung cancer [136].

#### Circadian rhythms and oxidative stress

Recent studies have indicated that the hypoxic response in cancer could be directly controlled by the circadian rhythm Clock/Bmal1 [137]. In a similar way, blood oxygen levels present daily rhythms influenced by clock genes [138]. Metabolic dysregulation in cancers may results of disruption of Bmal1 in a hypoxic-dependent way [139]. Considerable evidence connects circadian disruption with hormone-dependent diseases, such as breast and prostate cancers. One of the main factors is melatonin, a hormone produced by the pineal gland in a circadian manner to regulate sleep [140]. In the mitochondria, melatonin is linked to the regulation of oxidative stress [141]. Melatonin stimulates the activity of glutathione peroxidase and glutathione reductase [142]. Moreover, melatonin directly regulates the mitochondrial respiratory chain, which modulates ATP production [141]. Furthermore, alteration of melatonin secretion by sleep disruption could increase ROS and RNS production [143].

#### Interaction between the WNT/β-catenin pathway and circadian rhythms (Fig. 4)

WNT/β-catenin pathway is the downstream target of the RORs control factors and has several putative Bmal1 clock-binding sites within its promoter [144]. Through such interactions, circadian genes can regulate cell cycle progression through the WNT pathway [145]. The WNT pathway can be inhibited by a Bmal1 knockdown [146]. Expression levels of WNT-related genes in wild-type mice are higher than those observed in Bmal1 knockdown mice [147]. Cell proliferation and cell cycle progression are controlled by Bmal1 through the activation of the canonical WNT/β-catenin pathway [148]. Bmal1 enhances β-catenin transcription, inhibits β-catenin degradation and downregulates GSK-3β activity [149]. Per2 degradation induced by β-catenin increases circadian disruption in the intestinal mucosa of ApcMin/+ mice [150].

**Fig. 4:**
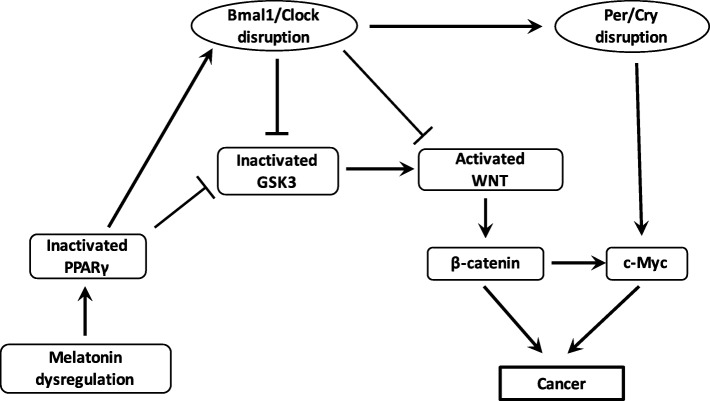
Interactions between PPARγ, WNT pathway and circadian rhythms in cancer. Dysregulation of melatonin and nocturin decreases the expression of PPARγ in cancer. Decreased PPARγ dysregulates Bmal1/Clock heterodimer. Decreased PPARγ expression directly activates the formation of the heterodimer Bmal1/Clock and β-catenin cytosolic accumulation but inhibits the activity of GSK3, the main inhibitor of the WNT/β-catenin pathway. Bmal1/Clock knockout also decreases GSK3 activity and activates the WNT/β-catenin pathway and its downstream gene c-Myc through the activation of the heterodimer Per/Cry. The activation of the WNT/β-catenin pathway by the cytosolic accumulation of the β-catenin and the activation of c-Myc lead to cancer initiation (oxidative stress and chronic inflammation)

In physiological conditions, the core circadian genes work in accurate feedback loops and keep the molecular clockworks in the SCN. They allow the regulation of peripheral clocks [121]. Per1 and Per2 maintain cells circadian rhythm and regulate cell-related genes expression, including c-Myc, so as to sustain the normal cell cycle [151]. Levels of mRNAs and proteins of circadian genes oscillate throughout the 24 h’ period [121].

#### Action of PPARγ on the circadian rhythms (Fig. 4)

PPARγ acts directly with the core clock genes and presents diurnal variations in liver and blood vessels [27]. In mice, impaired diurnal rhythms are induced by the inhibition of PPARγ [152]. PPARγ agonists can regulate Bmal1 and then the formation of the heterodimer Clock/Bmal1 [27] and can target Rev-Erb [153]. Downregulation of the clock-controlled gene Nocturin inhibits PPARγ oscillations in the liver of mice fed on a high-fat diet. In physiological conditions, nocturin binds PPARγ to improve its transcriptional activity [154]. PPARγ deletion alters the circadian function of 15-Deoxy-D 12,14-prostaglandin J2 (15-PGJ2) [152]. The partner of PPARγ, RXR, interacts with Clock protein in a ligand-dependent manner and then blocks Clock/Bmal1 heterodimer formation and transcriptional activity [155]. PPARγ acts on the mammalian clock to control energy metabolism. Circadian metabolism is directly controlled by PPARγ [152]. Retinoic acid receptor-related orphan receptor gamma t (ROR gammat) is considered as a major transcriptional factor for Th17 differentiation [156]. PPARγ can influence the function of Th cells clones [157]. PPARγ agonists inhibits Th17 differentiation through the inhibition of ROR gammat induction [158]. CD4+ T cells fail to express ROR gammat under the action of PPARγ agonists [159].

### Curcumin in cancers

#### Curcumin, an angiogenesis and metastasis inhibitor (Fig. 5)

Numerous studies have shown that curcumin inhibits the precursors of angiogenesis in cancers [160]. Chemical agonists of curcumin also induces the suppression of angiogenesis [16]. Curcumin downregulates the osteopontin (OPN, a secreted phosphoprotein 1)-induced cells leading to the downregulation of VEGF signaling and then the NF-ϰB/AT-4-dependent pathway [161]. Moreover, tetrahydrocurcumin, an analog of curcumin, can decrease the expression of several targets, such as COX-2, VEGF, MMP-9 [162].

**Fig. 5:**
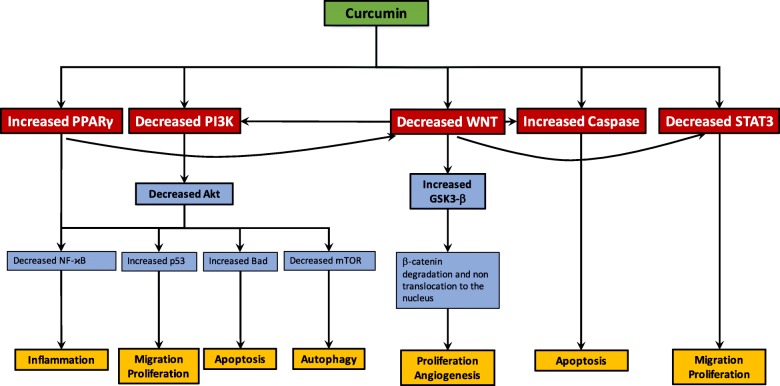
Curcumin actions on the WNT pathway in cancer therapy. Curcumin modulates cancer progression through the regulation of several signaling pathways. Attachment of ligands to their corresponding receptors leads to the activation of downstream pathways, including PI3K, STAT, caspase. These signaling pathways have a major role in cell survival, proliferation, apoptosis, angiogenesis, migration and metastasis. The decrease of Akt pathway by curcumin leads to the activation of p53 signaling and Bad-mediated apoptotic pathway contributing to cancer cell survival. Moreover, the downregulation of Akt pathway is associated with the inhibition of NF-ϰB signaling pathway, responsible for the inflammation. By decreasing WNT pathway, curcumin leads to the activate GSK-3β activity which induces β-catenin phosphorylation and then its degradation. The inhibition of the WNT pathway is associated with the control of proliferation and angiogenesis. The increase of caspase pathway by curcumin leads to apoptosis whereas curcumin decreases the STAT3 signaling pathway to counteract migration and proliferation. The activation of PPARγ by curcumin leads to the downregulation of the WNT pathway and the control of inflammation. WNT pathway downregulation results in the decrease of PI3K and STAT3 signaling pathways but the increase of caspase

The complex process of metastasis involves numerous alterations and degradations of the ECM by MMP which leads to the over-expression of chemokine receptors, inflammation and then angiogenesis. Curcumin inhibits cell migration in colorectal cancer by inhibiting MMP-9 activity and NF-ϰB and in the same time by activating AP-1 [163]. Furthermore, in prostatic cancer, cell migration is inhibited by curcumin which acts by decreasing inflammatory environment through the abolition of pro-inflammatory cytokines [164].

#### Curcumin inhibits the canonical WNT/β-catenin pathway (Fig. 5)

Cell-cycle is arrested in G2/M step in medulloblastoma cells by using curcumin inhibiting the WNT/β-catenin pathway [165]. Curcumin directly stimulates GSK-3β activity leading to the loss of nuclear β-catenin level and thus the inactivation of cyclin D1. In osteosarcoma cells, curcumin analogs disrupt β-catenin nuclear translocation [166]. In the 43-existing analog of curcumin, 6 analogs present a more potent activity compared to curcumin in the inhibition of the WNT pathway. Curcumin downregulates the 12–0-tetradecanoylphorbol-13-acetate (TPA)-induced WNT pathway in xenograft mice models [167]. Curcumin and its analog (CHC007) inhibit β-catenin/TCF/LEF complex in both colon, gastric, intestinal cancer cells [168]. Moreover, curcumin increases GSK-3β mRNA level in DAOY cells of medulloblastoma and thus downregulates the WNT/β-catenin pathway [169]. Through the inhibition of the WNT/β-catenin pathway, curcumin inhibits cyclin D1 and participates to the repression of the development and proliferation of gliomas [169].

#### Curcumin inhibits Akt pathway (Fig. 5)

In Burkitt’s lymphoma cells, curcumin increases radiation-induced apoptosis through the inhibition of the PI3K/Akt pathway [170]. Moreover, the efficacy of curcumin is equivalent to Akt-specific inhibitors, such as LY294002 for PI3K and SH-5 for Akt. In prostatic cancer, curcumin directly targets the PI3K/Akt pathway [171]. The combination of curcumin with the PI3K-specific inhibitor LY294002 has shown a beneficial effect by increasing the inhibition of Bcl-2 protein [172].

#### Curcumin stimulates PPARγ (Fig. 5)

Few studies have reported the PPARγ agonist role of curcumin. However, curcumin is known to induce apoptosis, and to inhibit cell proliferation and inflammation by stimulating PPARγ [173]. Through the activation of PPARγ, curcumin inhibits tumor growth by downregulating cyclin D1 and EGFR expression [174]. In parallel, the inhibition of EGFR signaling by curcumin is associated with the increase of PPARγ expression in hepatic stellate cell of rats [175].

#### Curcumin and inflammation (Fig. 6)

Several studies have suggested that curcumin can alleviate oxidative stress and inflammation through the Nrf2-keap1 pathway [176]. In various cancer cells, curcumin decreases pro-inflammatory signaling related and then inhibits the activation of TNF-α [177]. Moreover, curcumin decreases the release of different interleukins by acting on the NF-κB pathway. Curcumin acts as a stress response mimetic which leads to many compounds of the protein homeostasis network [178]. Curcumin present several clinical therapeutic potentials in many type of cancer cells [179]. Curcumin acts as a modulator of cellular pathways on multiple targets which control tumor growth, angiogenesis, metastasis, inflammation, and apoptosis [180].

**Fig. 6:**
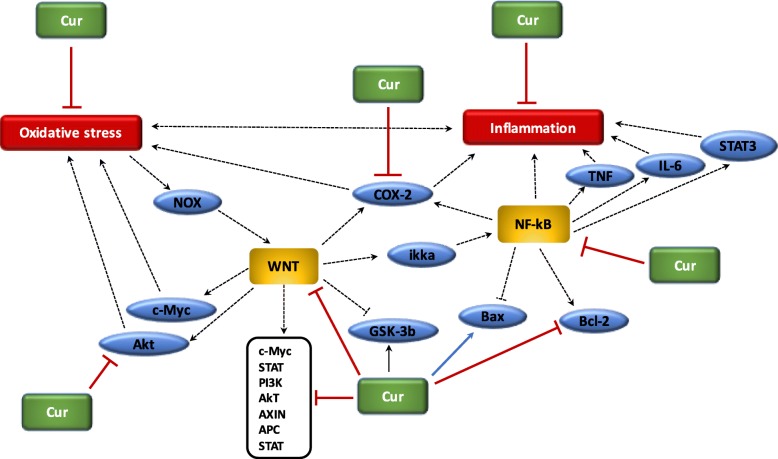
Beneficial role of curcumin in cancer. (1) Curcumin reduces oxidative stress; (2) Curcumin reduces chronic inflammation; (3) Curcumin inhibits Akt pathway activity; (4) Curcumin downregulates WNT pathway and its target genes, inhibits Bcl-2 and activates GSK-3beta; (5) Curcumin inhibits NF-ϰB and COX-2

Cancer process is responsible for the activation of the NF-κB pathways leading to the over-expression of pro-inflammatory factors, including COX-2, iNOS, cytokines, TNF-α [181]. Curcumin presents an anti-proliferative role thought the inhibition of the NF-κB and its downstream genes, such as p53, VEGF, Bcl-2, COX-2, NOS, cyclin D1, TNF-α, interleukins and MMP-9) [182]. Curcumin is considered as an interesting therapeutic way in melanoma cells through the inhibition of NF-ϰB, STAT3 and COX-2 pathways [183]. Curcumin inhibits pro-inflammatory cytokines CXL1 and CXCL2 to decrease the formation of prostatic and breast metastases [184]. Curcumin inhibits the HPV-16-induced viral oncogenesis in oral tumor cell lines. Curcumin induces the blockage of the DNA-binding capacity of NF-ϰB through the alteration of its subunits from p50/p50 to p50/p65. The suppression of the NF-ϰB activity by curcumin is associated with the downregulation of AP-1 families of transcription factors [185]. Moreover, in mouse melanoma cells, curcumin induces the activation of the caspase-3 and the dose-dependent inhibition of the NF-ϰB activity and thus the inhibition COX-2 and cyclin-D1 expression [186].

#### Curcumin and oxidative stress (Fig. 6)

Recent findings have shown that curcumin presents anti-inflammatory effects mediated by the inactivation of the NF-ϰB pathway [187], but rather on its oxidized products [188]. Oxidative metabolites of curcumin inhibit IKK. Treatment with *N*-acetylcysteine, a biosynthetic precursor of glutathione (GSH), the effect of curcumin was decreased, probably due to GSH-mediated scavenge and thus inactivating of curcumin-derived electrophile [188]. Oxidative stress, observed in cancer process, is based on the hypothesis of chronic inflammation [189]. The recent anti-tumorigenic role of curcumin in human leukemic cells may confirmed the presence of oxidized curcumin metabolites [188]. Indeed, curcumin is known to be a natural component presenting antioxidant effects [190]. Due to its chemical structure, curcumin is indeed a scavenger of ROS and RNS [191]. In addition, curcumin is a lipophilic compound, which makes it an efficient collector of peroxyl radicals. Curcumin controls the activity of GSH, catalase, and SOD enzymes activated in the neutralization of free radicals. Curcumin decreases ROS-generating enzymes such as lipoxygenase/cyclooxygenase and xanthine hydrogenase/ oxidase [192]. Inhibition of oxidative-stress induced DNA damage has been shown in curcumin treated mouse fibroblast cells [193]. In the leukemic cells, curcumin directly targets Nrf2 to downregulate ROS production [194].

#### Curcumin and circadian rhythms

Few studies have investigated the role of curcumin with circadian clock in cancers [195]. However, Bmal1 appears to be a target of curcumin through the stimulation of PPARγ [27, 196]. Curcumin activates sirtuin 1 (SIRT1) which regulates circadian rhythms. SIRT1 indirectly modulates circadian clock through the downregulation of NF-ϰB [197], the inhibition of nuclear localization of Per2 [198] and the binding to Clock/Bmal1 [199].

### Relevance of “chronotherapy” in cancer clinical therapy

The numerous interactions between clock dysregulation and cancer underline the interest of circadian therapeutic actions [26]. The temporal peak of cell activity could be targeted by pharmacological drugs used at an optimal time of day. Few studies have focused on the potential role of WNT and PPARγ with circadian clocks in cancer development. Nevertheless, interest in the association between PPARγ agonists and melatonin in cancer therapy is not new [200]. In cultured cells, the addition of melatonin with a PPARγ agonist (such as troglitazone) is associated with a significant reduction in cell numbers [201]. Moreover, other studies have shown a potent apoptotic effect of a combination of melatonin with PPARγ agonists in breast cancer cells [202, 203]. In parallel, recent studies have shown that melatonin could inhibit WNT pathway expression [204, 205].

In mouse ovaries, melatonin administration protects against ROS production and mitochondrial damage [206]. In colorectal cancer, the combination of 5-fluorouracil and melatonin is associated with the inhibition of cell proliferation through suppression of the PI3K/Akt pathway, NF-ϰB pathway and nitric oxide synthase signaling [207]. Moreover, melatonin inhibits GSK3-β to stop invasion in breast cancer cells [208]. The link between carcinogenesis and the circadian clock remains complex and difficult to unravel. Strong evidence suggests the involvement of the circadian clock in cancer development. Numerous molecular pathways are dynamically circadian, such as the WNT/β-catenin pathway and PPARγ. Thus, the time at which these pathways are targeted may be critical. Curcumin, by acting as PPARγ agonists and focusing on the WNT/β-catenin pathway, should be used in concordance with the circadian clock genes, and therefore administered at the optimum time of day. Further studies should focus on the importance of the day/night cycle in cancer therapy and the circadian profiles of cancer cells.

## Conclusion

Cancers are associated with chronic inflammation, oxidative stress and circadian clock disruption. The over-activation of the WNT/β-catenin pathway increases these pathological phenomena. In cancers, the WNT/β-catenin pathway is upregulated whereas PPARγ is downregulated. These two signaling pathways act in opposing manners and this could explain their unidirectional profile observed in cancers. Moreover, in cancers, the disruption of circadian clock leads to the increase of the WNT/β-catenin pathway and to decrease of PPARγ expression. The strongly link between circadian rhythms, chronic inflammation and oxidative stress appears to be a major mechanism underlying cancers. The use of curcumin, which acts as PPARγ agonists, could be interesting in the reduction of both chronic inflammation and oxidative stress, and in the control of circadian clock by inhibiting the WNT/β-catenin pathway. Due to the considerable impact of cancers on mortality and morbidity rates worldwide, it would appear of the utmost importance to better understand the action of curcumin in cancers and particularly its role in the inhibition of the major signaling system known as the WNT/β-catenin pathway.

## Data Availability

Not applicable.

## Abbreviations

APC: Adenomatous polyposis coli
Bmal1: Brain and muscle aryl-hydrocarbon receptor nuclear translocator-like 1
CK1: Casein kinase 1
Clock: Circadian locomotor output cycles kaput
COX-2: Cyclooxygenase-2
CRD-BP: Coding Region Determinant-Binding Protein, an RNA-binding protein
CRs: Circadian rhythms
Cry: Cryptochrome
FZD: Frizzled
GSK-3β: Glycogen synthase kinase-3β
IϰB-α: Nuclear factor of kappa light polypeptide gene enhancer in B-cells inhibitor, alpha
LRP 5/6: Low-density lipoprotein receptor-related protein 5/6
MAPK: Mitogen-activated protein kinases
NF-ϰB: Nuclear factor ϰB
NOX: NADPH oxidase
Per: Period
PI3K-Akt: Phosphatidylinositol 3-kinase-protein kinase B
PPARγ: Peroxisome proliferator-activated receptor gamma
RORs: Retinoid-related orphan receptors
ROS: Reactive oxygen species
TCF/LEF: T-cell factor/lymphoid enhancer factor
TNF-α: Tumor necrosis factor alpha
βTrCP: Beta-transducin repeat-containing protein
